# Chios Mastic in Well‐Controlled Hypertension: Findings From the HYPER‐MASTIC Randomized Controlled Trial

**DOI:** 10.1002/prp2.70322

**Published:** 2026-10-15

**Authors:** Ioanna Panagiota Kalafati, Anastasia‐Areti Kyriazopoulou‐Korovesi, Evangelia Mentsiou‐Nikolaou, Maria Papaioannou, Christina Konstantaki, Lasthenis Angelidakis, Despoina D. Gianniou, Eleni V. Mikropoulou, Panagiota Giardoglou, Dimitrios Valakos, Giannis Vatsellas, HYPER‐MASTIC Consortium, Ioanna Andreadou, Efstathios Manios, Maria Halabalaki, Ioannis P. Trougakos, George V. Dedoussis, Kimon Stamatelopoulos

**Affiliations:** ^1^ Department of Nutrition and Dietetics, School of Health Science and Education Harokopio University of Athens Athens Greece; ^2^ Department of Clinical Therapeutics, School of Medicine National and Kapodistrian University of Athens Greece; ^3^ Department of Cell Biology and Biophysics, Faculty of Biology National and Kapodistrian University of Athens Athens Greece; ^4^ Division of Pharmacognosy and Natural Products Chemistry, Department of Pharmacy National and Kapodistrian University of Athens Athens Greece; ^5^ Multi‐Omics Laboratory, Center of Systems Biology Biomedical Research Foundation of the Academy of Athens Athens Greece; ^6^ Laboratory of Pharmacology, Faculty of Pharmacy National and Kapodistrian University of Athens Athens Greece

**Keywords:** Chios mastic, hypertension, randomized controlled trial, residual cardiovascular risk, transcriptomics, vascular function

## Abstract

Residual cardiovascular risk persists in patients with adequately controlled hypertension, driven partly by inflammation, oxidative stress and endothelial dysfunction. Chios mastic ( *Pistacia lentiscus* var. *Chia*) has shown antihypertensive, antioxidant and vascular effects in experimental and clinical settings. This trial evaluated whether Chios mastic supplementation confers additional cardiovascular benefits in well‐controlled hypertensive individuals. In this double‐blind, randomized, placebo‐controlled trial, 106 adults with stable, well‐controlled hypertension and variable cardiovascular risk, based on SCORE2, received low‐dose Chios mastic (1500 mg/day), high‐dose Chios mastic (2800 mg/day) or placebo for 3 months. Primary endpoints were changes in 24‐h ambulatory systolic and diastolic blood pressure. Secondary endpoints included vascular function, oxidative stress and proteasome activity in peripheral blood mononuclear cells and red blood cells. Transcriptomic profiling was performed in a predefined high‐risk subgroup. Chios mastic supplementation was well tolerated, with high adherence confirmed by dose‐dependent increases in plasma masticadienonic and isomasticadienonic acids. No significant between‐group differences were observed in 24‐h systolic or diastolic blood pressure after the intervention. Oxidative stress markers and proteasome activity remained unchanged across groups. In high‐risk participants, mastic supplementation induced distinct transcriptomic changes, particularly in pathways related to endothelial junction organization and vascular integrity. Overall, in adults with well‐controlled hypertension and low baseline cardiovascular risk, Chios mastic supplementation did not lower blood pressure over 3 months, likely reflecting a ceiling effect. Transcriptomic findings suggest biologically meaningful vascular effects in individuals with higher residual cardiovascular risk, supporting future investigation in higher‐risk populations.

## 1 Introduction

Hypertension remains the most prevalent risk factor for cardiovascular disease (CVD) and a major public health challenge [1, 2]. Although optimal management of blood pressure (BP) and other metabolic parameters reduces this burden, a residual cardiovascular risk of 20%–40% persists in well‐controlled hypertensive patients [3, 4, 5]. This residual risk is attributed to chronic inflammation, subclinical conditions, and genetic predisposition affecting pathways of lipid metabolism [6, 7], the renin–angiotensin system [8, 9], endothelial function, and inflammation [10]. Epigenetic factors, including microRNA‐21, have also been implicated in hypertensive vascular remodeling [11, 12].

Oxidative stress contributes to CVD progression by promoting endothelial dysfunction, vascular inflammation and arterial stiffness [13]. Reactive oxygen species (ROS) damage cellular components, whereas the proteasome maintains protein homeostasis by degrading oxidized or misfolded proteins; impaired proteasome function has been linked to increased oxidative stress and inflammation [14]. ROS levels and proteasome activity in peripheral blood mononuclear cells (PBMCs) and red blood cells (RBCs) therefore provide insight into these mechanisms.

Chios mastic gum, the resinous exudate of *Pistacia lentiscus* var. *Chia*, contains masticadienonic (MNA) and isomasticadienonic acid (IMNA) as its most characteristic triterpenes [15, 16]. Beyond its traditional gastroprotective use [17], mastic exhibits antioxidant, anti‐inflammatory, and cardioprotective properties [15, 18], and recent evidence suggests an antihypertensive effect in human and animal studies [19, 20, 21]. Kontogiannis et al. first reported an acute antihypertensive effect of mastic in humans, with preliminary findings implicating proteostatic and antioxidant mechanisms [19].

We hypothesized that Chios mastic might reduce residual cardiovascular risk in well‐controlled hypertensive patients. The HYPER‐MASTIC double‐blind, randomized, placebo‐controlled trial was therefore designed primarily to assess the superiority of two doses of Chios mastic on BP and vascular parameters compared to placebo, and secondarily to evaluate antioxidant, anti‐inflammatory, and proteostatic effects through ROS and proteasome activity in PBMCs and RBCs. RNA sequencing was used to identify mastic‐induced transcriptomic changes, whereas microRNA‐21 levels were assessed in a parallel report [12].

## 2 Materials and Methods

### 2.1 Trial Design and Ethics

The HYPER‐MASTIC trial (https://www.hyper‐mastic.eu) was a 3‐month, randomized, double‐blind, placebo‐controlled, parallel‐group intervention conducted at the Department of Clinical Therapeutics of “Alexandra” General Hospital (National and Kapodistrian University of Athens, Greece) from 04/2022 to 02/2024. All participants provided written informed consent. The trial adhered to the Declaration of Helsinki and ICH Good Clinical Practice guidelines and was approved by the Harokopio University Ethics Committee (35‐21/12/2021) and the Scientific Board of Alexandra Hospital (8/23‐12‐2021). Chios mastic was investigated as a nutraceutical supplement in this study and is not labeled by the U.S. Food and Drug Administration for the treatment of hypertension or cardiovascular risk reduction.

### 2.2 Participants and Randomization

Sample‐size calculation was based on pilot data from Kontogiannis et al. [19]; accounting for a 15% dropout per group (*n* = 30), 105 participants provided > 90% power to detect changes in 24‐h systolic (SBP) and diastolic blood pressure (DBP). Eligible participants were 40–80 years old, of either sex, with stable, well‐controlled arterial hypertension confirmed by 24‐h ambulatory BP monitoring for at least 2 months. Exclusion criteria included recent acute myocardial infarction or heart failure, stroke, mastic allergy, use of antioxidant supplements, active infection, autoimmune disease or cancer. Computer‐generated randomization in SPSS (v24) allocated participants to low‐dose mastic (1500 mg/day), high‐dose mastic (2800 mg/day) or placebo. Personnel involved in the enrollment and assessment of the patients had no access to the allocation sequence. Cardiovascular risk was assessed using SCORE2. A predefined high‐risk subgroup (*n* = 59) was identified comprising participants meeting at least one of: CRP ≥ 2 mg/L, ≥ 10 years post‐menopause, age ≥ 65 years, chronic renal failure, DBP ≥ 90 mmHg or SBP ≥ 140 mmHg [22, 23, 24, 25].

### 2.3 Blinding and Intervention

Both participants and study personnel remained blinded throughout, with randomization codes revealed only after trial completion. The low‐dose group received two 750‐mg tablets daily and the high‐dose group received four 700‐mg tablets daily; the placebo arm was correspondingly randomized into two‐ or four‐tablet regimens to mirror the mastic schedule. Tablets, manufactured by IASIS Pharmaceuticals Hellas S.A. (Athens, Greece), were identical in size and shape, with placebo tablets sprayed with mastic scent to preserve blinding. The 2800 mg/day dose was selected based on Kontogiannis et al. [19].

### 2.4 Study Protocol and Visit Schedule

The protocol comprised four study visits over a 3‐month period. The baseline visit (V0) included assessment of eligibility, medical history, current medication, anthropometry, lifestyle evaluation, comprehensive BP, vascular and cardiac assessment, and blood sampling. Two interim visits at Months 1 (V1) and 2 (V2) were performed for re‐assessment of office BP and vascular function, blood sampling, supplement re‐supply and adherence evaluation. The final visit at Month 4 (V3) included a comprehensive re‐assessment mirroring the baseline evaluation, including lifestyle re‐evaluation. Between visits, participants were contacted biweekly by site staff for adherence checks and adverse event monitoring. In total, measurements at four time points enabled close longitudinal monitoring throughout the 3‐month intervention.

### 2.5 Anthropometric and Lifestyle Assessments

Body weight (kg) and height (m) were measured by trained staff using calibrated equipment, and body mass index (BMI; kg/m^2^) was calculated. Lifestyle information was collected by trained dietitians at the baseline and final visits. Participants were instructed to maintain their habitual dietary and physical activity patterns throughout the 3‐month intervention. To verify adherence to this instruction, dietary habits were assessed using (a) a validated 76‐item food frequency questionnaire covering the typical intake over the preceding period [26], and (b) three 24‐h dietary recalls (two on weekdays and one on a weekend day) to capture day‐to‐day variability [27]. Physical activity was evaluated using the short form of the International Physical Activity Questionnaire (IPAQ) [28], with weekly metabolic equivalent (MET) minutes calculated according to the standard scoring protocol. Smoking status, pack‐years, alcohol consumption, and current medication were also recorded at every visit.

### 2.6 Blood Pressure, Vascular and Cardiac Assessment

Twenty‐four‐hour ambulatory BP monitoring (Holter) and a comprehensive battery of vascular and cardiac measurements were performed at baseline (V0) and at the final visit (V3). Office BP and a focused vascular assessment (FMD, PWV, central pressures) were repeated at the interim visits (V1 and V2). All assessments were performed in a quiet, temperature‐controlled room after at least 10 min of supine rest, with participants instructed to abstain from caffeine, smoking, and vigorous exercise for at least 12 h prior to the visit.

Twenty‐four‐hour ambulatory peripheral and central (aortic) BP were recorded using a Mobil‐O‐graph monitor (IEM, Aachen, Germany), generating 24‐h, daytime and night‐time profiles for SBP, DBP, central SBP (cSBP), central DBP (cDBP), and pulse wave velocity (PWV24hr). Baroreceptor function was assessed using a Finometer device (Finapres Medical Systems, Enschede, the Netherlands). Endothelial function was evaluated by brachial artery flow‐mediated dilatation (FMD), expressed as the percentage change in arterial diameter following 5 min of forearm cuff occlusion. Arterial stiffness was evaluated using carotid–femoral PWV, the gold‐standard method based on the calculation of carotid and femoral pulse pressures and the transit time between them (Complior, Alam Medical, Saint‐Quentin‐Fallavier, France). Imaging data for PWV analysis were processed using the Brachial Analyzer for Research software (Medical Imaging Applications LLC, Coralville, IA, USA). Wave reflection and aortic pressure waveforms were assessed by applanation tonometry. Atherosclerotic burden was assessed by ultrasound examination of the carotid and femoral arteries to measure intima—media thickness (IMT) and detect atherosclerotic plaques. Peripheral arterial disease was screened using the ankle–brachial index. Cardiac function was evaluated by transthoracic echocardiography. All vascular and cardiac procedures were performed by a single researcher with extensive expertise to minimize inter‐observer variability; physical examinations, medical history‐taking, and clinical data collection were performed by an internal medicine physician.

### 2.7 Biological Samples and Laboratory Analyses

Blood samples were drawn at every visit. Plasma, serum and buffy coat were stored at −80°C. PBMCs and RBCs were isolated by density‐gradient centrifugation. ROS levels were measured using CM‐H2DCFDA fluorometric detection (Invitrogen, Carlsbad, CA, USA), and proteasome chymotrypsin‐like activity was measured by hydrolysis of the fluorogenic peptide Suc‐Leu‐Leu‐Val‐Tyr‐AMC (Enzo Life Sciences, Farmingdale, NY, USA). Plasma MNA and IMNA were quantified by ultra‐performance liquid chromatography coupled to high‐resolution mass spectrometry (Acquity H‐class UPLC coupled to AB Sciex TripleTOF 5600+; Waters, Milford, MA, USA; AB Sciex, Marlborough, MA, USA) as previously described [29]. RNA was isolated from whole blood (Monarch Total RNA, New England Biolabs, Ipswich, MA, USA) and RNA sequencing was performed at the Greek Genome Center (Biomedical Research Foundation of the Academy of Athens, Athens, Greece). Differential gene expression analysis was performed with DESeq2 [30] and functional enrichment with EnrichR [31]; transcriptomic analysis was restricted to the high‐risk subgroup, which was expected to display heightened inflammatory activity. Detailed protocols for sample isolation, ROS/proteasome assays, MNA/IMNA quantification and RNA sequencing are provided in Supporting Information.

### 2.8 Adherence Assessment

Adherence to the intervention was rigorously monitored throughout the study. Participants recorded daily supplement intake in a diary and returned both the diary and supplement containers at each visit. Biweekly telephone follow‐ups by trained dietitians assessed supplement use, adverse events, and major dietary changes using a 24‐h dietary recall, enabling appropriate guidance or, when necessary, exclusion from analysis. Following the intervention and unblinding, adherence was further verified objectively by measuring plasma MNA and IMNA concentrations; pre–post within‐group changes and between‐group comparisons were interpreted alongside the traditional adherence measures.

### 2.9 Statistical Analysis

All randomized participants who completed the 3‐month intervention were included in the subsequent analysis. Continuous variables are expressed as mean (SD) or median (interquartile range, IQR) according to the Kolmogorov–Smirnov test. Between‐group comparisons used independent *t*‐tests, Mann–Whitney *U*, one‐way ANOVA, or Kruskal–Wallis, as appropriate. Within‐group changes were assessed by paired *t*‐tests or Wilcoxon signed‐rank tests. Repeated‐measures ANOVA with Bonferroni post hoc correction was used to evaluate time‐by‐group interactions, adjusted for age and sex. Sensitivity analyses in the high‐risk group and in the participants with not known coronary artery disease (non‐CAD subgroup) were also performed. Analyses were performed in SPSS v27.0.1 (IBM Corp., Armonk, NY, USA) with *α* = 0.05.

## 3 Results

### 3.1 Participant Characteristics

The supplement was well tolerated, with no serious adverse events. Baseline characteristics for the placebo (*n* = 31), low‐dose (*n* = 25), and high‐dose (*n* = 25) groups are shown in Table 1. Groups were comparable on most variables, except for younger age in the low‐dose group (*p* = 0.021) and a higher frequency of family history of arterial hypertension in the mastic groups (*p* = 0.022). Other CVD risk factors did not differ between groups. The CONSORT flow diagram is shown in Figure 1.

**TABLE 1:** Baseline characteristics of participants by treatment group.

Variable	Placebo (*N* = 31)	Low dose (*N* = 25)	High dose (*N* = 25)	*p*
Age (years)	63.74 ± 6.20*	58.12 ± 7.22*	62.44 ± 9.21	***0.021***
Female sex (%)	51.6	28	40	0.20
BMI (kg/m^2^)	28.21 ± 4.31	33.72 ± 18.57	29.36 ± 4.89	0.33
Years since diagnosis of AH	10 (19)	7 (10)	10 (16)	0.29
Family history of AH (% Yes)	51.6	76	84	***0.022***
Family history of CAD (% Yes)	35.5	44	36	0.78
Family history of hyperlipidemia (% Yes)	45.2	48	36	0.67
DM2 (% Yes)	22.6	20	24	0.94
CAD (% Yes)	9.7	20	4	0.18
Hyperlipidemia (% Yes)	71	64	68	0.86
MET min per week	2022.96 ± 626.69	1489.45 ± 1101.57	1785 ± 1853.12	0.52
Smokers (%)	30	20	20	0.59
Pack‐years	10 (20)	8 (25)	2 (38)	0.97
Energy intake (kcal)	1574.44 ± 530.95	1428.29 ± 342.16	1382.44 ± 614.56	0.63

*Note:* Values are presented as mean (SD) for normally distributed variables, as median (interquartile range, IQR) for non‐normally distributed variables, or as relative frequencies for categorical variables. *p*‐values were calculated with one‐way ANOVA or Kruskal–Wallis tests according to the normality of distribution. Significant values (*p* ≤ 0.05) shown in bold italic.

Abbreviations: AH, arterial hypertension; BMI, body mass index; CAD, coronary artery disease; DM2, diabetes mellitus type 2; MET, metabolic equivalent.

* Significant pairwise differences based on Bonferroni post hoc test.

**FIGURE 1:**
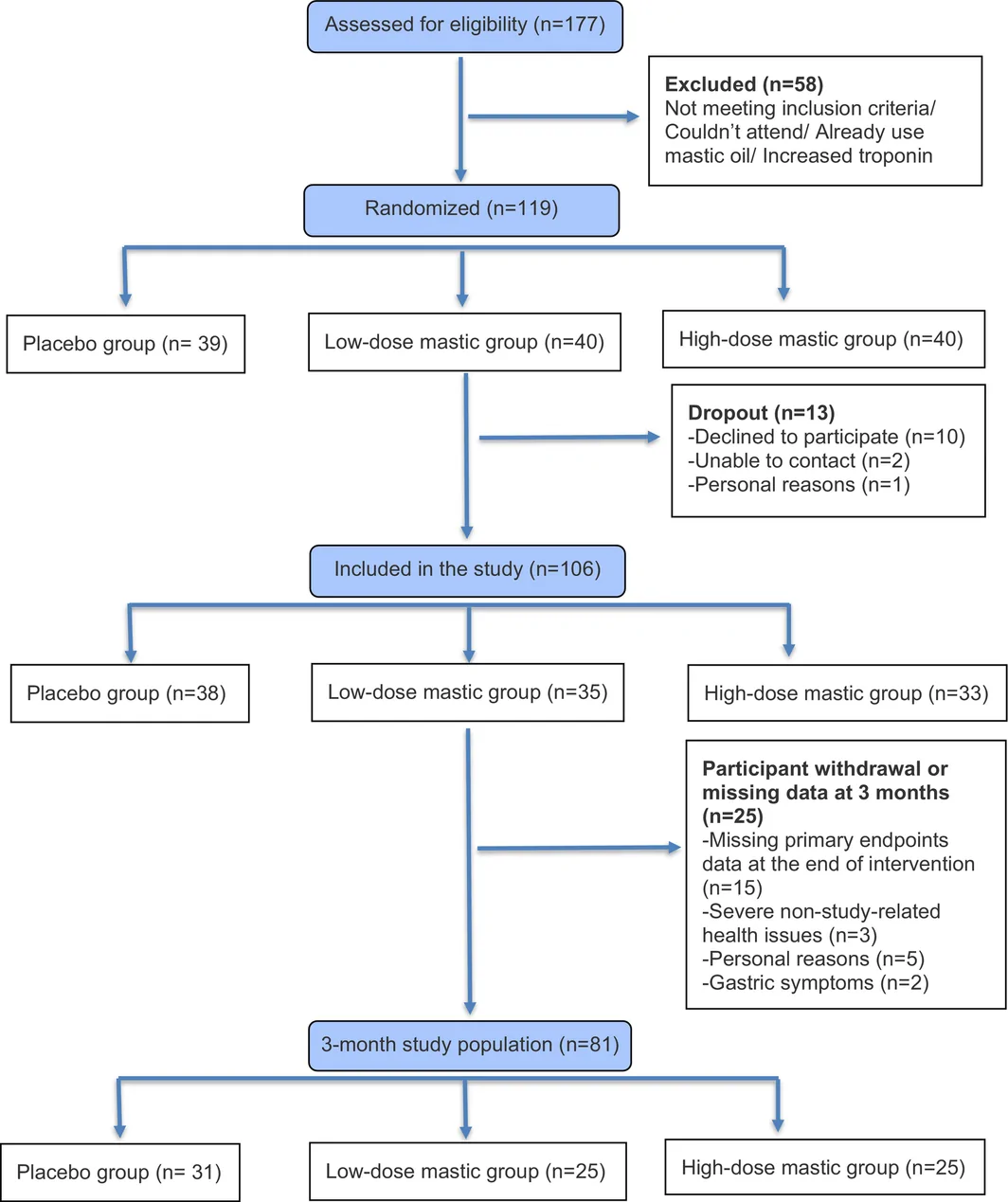
CONSORT flow diagram of the HYPER‐MASTIC study. Flow diagram illustrating the progress of participants through the phases of the double‐blind, randomized, placebo‐controlled HYPER‐MASTIC trial, including enrollment, randomization, total number of participants included and final number of volunteers who completed the 3‐month intervention. Participants who withdrew or lacked follow‐up measurements for the primary endpoints (*n* = 25) were excluded from analysis.

### 3.2 Adherence to Intervention

At baseline, MNA and IMNA levels did not differ between groups. After 3 months, both metabolites differed significantly across groups: levels remained unchanged in the placebo group, increased in the low‐dose group, and rose approximately three‐fold further in the high‐dose group (Table S1, Figure S1). Time‐by‐intervention analyses confirmed significant differences between mastic and placebo arms, indicating high adherence to the prescribed regimen.

### 3.3 Twenty‐Four‐Hour Ambulatory Blood Pressure (Primary Endpoint)

At baseline, PWV24hr in the low‐dose group was significantly lower than in the high‐dose group (Table 2). After 3 months, the low‐dose group showed a numerical increase in SBP24hr and statistically significant within‐group increases in PWV24hr, PWVday, cSBPday, cDBPday, SBPnight, PWVnight, and cSBPnight; however, none of these outcomes showed a significant time‐by‐group interaction (*p* > 0.05). A significant time‐by‐group interaction was observed for cSBP24hr (*p* = 0.017), reflecting a trend toward an increase in the low‐dose group relative to placebo and high‐dose groups (Table 2).

**TABLE 2:** Total 24‐h Holter monitoring measurements pre‐ and post‐intervention.

Variable	Time	Placebo (*N* = 31)	Low dose (*N* = 25)	High dose (*N* = 25)	P groups	P time*group
SBP24hr (mmHg)	Baseline	121.71 ± 9.45	122.88 ± 7.80	123.44 ± 7.88	0.74	0.18
	3rd month	122.42 ± 8.79	126.4 ± 8.06	124.12 ± 7.34	0.20	
	P time	0.70	***0.002***	0.59		
DBP24hr (mmHg)	Baseline	75.74 ± 8.67	76.52 ± 7.72	77.08 ± 8.29	0.83	0.17
	3rd month	74.87 ± 7.55	77.56 ± 7.95	75.96 ± 7.45	0.43	
	P time	0.452	0.164	0.51		
PWV24hr (m/s)	Baseline	8.82 ± 1.04	8.15 ± 0.93*	8.83 ± 1.30*	***0.03***	0.49
	3rd month	8.87 ± 0.98	8.30 ± 1.02	9.10 ± 1.51	0.05	
	P time	0.39	***0.001***	0.22		
cSBP24hr (mmHg)	Baseline	124.23 ± 9.59	121.12 ± 23.72	127 ± 9.20	0.35	***0.017***
	3rd month	124.23 ± 9.59	130.24 ± 8.08	127.92 ± 11.8	0.22	
	P time	0.462	0.052	0.645		
cDBP24hr (mmHg)	Baseline	76.84 ± 8.79	78.16 ± 7.63	78.71 ± 8.70	0.72	0.093
	3rd month	76.71 ± 7.24	79.84 ± 7.83	77.67 ± 7.50	0.30	
	P time	0.93	0.059	0.48		

*Note:* The *p*‐value for time was calculated using paired‐sample *t*‐tests to compare pre‐ and post‐intervention levels within each treatment group. Between‐group differences at each time point (P groups) were assessed using one‐way ANOVA, and the time×group interaction was evaluated using repeated‐measures ANOVA, adjusted for age and sex. Significant values (*p* ≤ 0.05) shown in bold italic.

Abbreviations: cSBP/cDBP, central systolic/diastolic blood pressure; DBP, diastolic blood pressure; PWV, pulse wave velocity; SBP, systolic blood pressure.

* Significant pairwise differences based on Bonferroni post hoc test.

Crucially, changes in 24‐h SBP and DBP—the primary endpoints—did not differ significantly between groups (ΔSBP and ΔDBP, *p* > 0.05; Figures S2 and S3), and within‐group paired comparisons showed no significant differences. Day‐ and night‐time ambulatory data are provided in Tables S2 and S3.

### 3.4 Office Blood Pressure, Biochemical and Vascular Measurements (Secondary Endpoints)

Office DBP increased by 2.47 mmHg in the high‐dose group after 3 months (*p* = 0.026). The placebo group showed a significant 4.92 mmHg reduction in central DBP (cDBPoffice) over time, with a significant time‐by‐group interaction (*p* = 0.028). FMD showed minimal variation across groups. Fasting glucose declined in the placebo group, with a significant time‐by‐group interaction (*p* = 0.017); other biochemical parameters were unchanged (Table 3, Table S4).

**TABLE 3:** Office blood pressure and vascular measurements: Changes from baseline to follow‐up visit.

Variable	Time	Placebo (*N* = 31)	Low dose (*N* = 25)	High dose (*N* = 25)	P groups	P time*group
SBPoffice (mmHg)	Baseline	122.5 ± 12.25	128.12 ± 10.49	126.68 ± 9.80	0.39	0.27
	3rd month	120 (17)	127.5 (18)	125 (17)	0.102	
	P time	0.45	0.92	0.71		
DBPoffice (mmHg)	Baseline	75.29 ± 8.58	79.16 ± 7.88	79.64 ± 8.88	0.29	0.316
	3rd month	73.6 ± 7.66	78.33 ± 9.36	77.17 ± 8.65	0.21	
	P time	0.38	0.39	0.81		
FMD (%)	Baseline	3.03 (3.28)	2.37 (2.66)	2.48 (2.01)	0.14	0.659
	3rd month	3.29 (3.36)	2.03 (1.08)	1.94 (2.31)	0.24	
	P time	0.28	0.57	0.43		
cSBPoffice (mmHg)	Baseline	111.74 ± 10.67	113.67 ± 10.09	112.04 ± 8.89	0.57	0.19
	3rd month	109.52 ± 9.48	114.16 ± 11.43	115.6 ± 13.29	0.33	
	P time	0.36	0.71	0.29		
cDBPoffice (mmHg)	Baseline	76.33 ± 7.14	76.79 ± 7.84	75.21 ± 8.81	0.24	***0.028***
	3rd month	71.41 ± 6.28	77.04 ± 9.64	75.25 ± 8.66	0.06	
	P time	***0.006***	0.98	0.91		
PWVoffice (m/s)	Baseline	9.02 (2.8)	9.3 (1.85)	10.3 (1.9)	0.18	0.75
	3rd month	9.52 ± 1.30	9.55 ± 1.25	10.35 ± 1.54	0.10	
	P time	***0.011***	0.66	0.81		

*Note:* The *p*‐value for time was calculated using paired‐sample *t*‐tests to compare pre‐ and post‐intervention levels within each treatment group. Between‐group differences at each time point (P groups) were assessed using one‐way ANOVA, and the time × group interaction was evaluated using repeated‐measures ANOVA, adjusted for age and sex. Significant values shown in bold italic.

Abbreviations: cDBPoffice, central office diastolic blood pressure; cSBPoffice, central office systolic blood pressure; DBPoffice, office diastolic blood pressure; FMD, flow‐mediated dilatation; PWVoffice, office pulse wave velocity; SBPoffice, office systolic blood pressure.

### 3.5 Oxidative Stress and Proteasome Activity

ROS and proteasome activity in PBMCs and RBCs did not differ significantly between groups at baseline or at 3 months. Within‐group analyses showed a significant increase in proteasome activity in PBMCs and RBCs in the low‐dose group (*p* = 0.019 and 0.026, respectively) and an increase in PBMC ROS in the high‐dose group (*p* = 0.03), but no significant time‐by‐group interactions emerged (Table S5). No significant correlations were found between changes in ROS/proteasome activity and changes in FMD or PWV (office and 24‐h ambulatory measurements).

### 3.6 High‐Risk and Non‐CAD Subgroup Analyses

Baseline characteristics of the high‐risk subgroup (*n* = 59) were largely comparable to the overall population, except for family history of hypertension (*p* = 0.027; Table S6). Findings were broadly consistent with the overall population: the low‐dose group showed significant within‐group increases in SBP24hr and PWV24hr, although no significant time‐by‐group interactions were observed (Table S7). cSBP24hr again showed a significant time‐by‐group interaction (*p* = 0.024), with the same increasing trend in the low‐dose group. cDBPoffice improved and PWVoffice increased in the placebo group, but these changes did not yield significant time‐by‐group interactions. Biochemical analyses showed no significant differences (Tables S8 and S9).

Moreover, a sensitivity analysis excluding participants with known coronary artery disease (non‐CAD subgroup, *n* = 72) yielded consistent results, with the exception of a significant time‐by‐group interaction for PWV24hr (*p* = 0.006), driven by lower baseline values in the low‐dose group (Tables S10–S13).

### 3.7 Transcriptomic Changes in High‐Risk Patients

To explore molecular effects beyond hemodynamic endpoints, RNA‐seq was performed on whole‐blood samples from high‐risk participants before and after the intervention; both mastic doses were merged for this analysis and compared with placebo. Transcriptomic analysis revealed significant gene‐expression changes following mastic supplementation compared with placebo, with a distinct set of up‐ and down‐regulated genes in the mastic group (Figure 2A,B). The Venn diagram showed that only 1% of genes (MIR93 and BCL2L10) were commonly differentiated, whereas 141 and 74 genes were uniquely altered in the mastic and placebo groups, respectively (Figure 2C). Pathway and functional enrichment analysis of upregulated genes revealed significant modulation of pathways related to tight junction organization and cellular adhesion (Figure 2D–F)—processes central to endothelial barrier integrity and vascular permeability, which are disrupted in hypertension. Several genes involved in cell–cell junction assembly and extracellular matrix interactions were differentially expressed in the mastic group, suggesting that mastic may exert protective vascular effects by enhancing endothelial stability.

**FIGURE 2:**
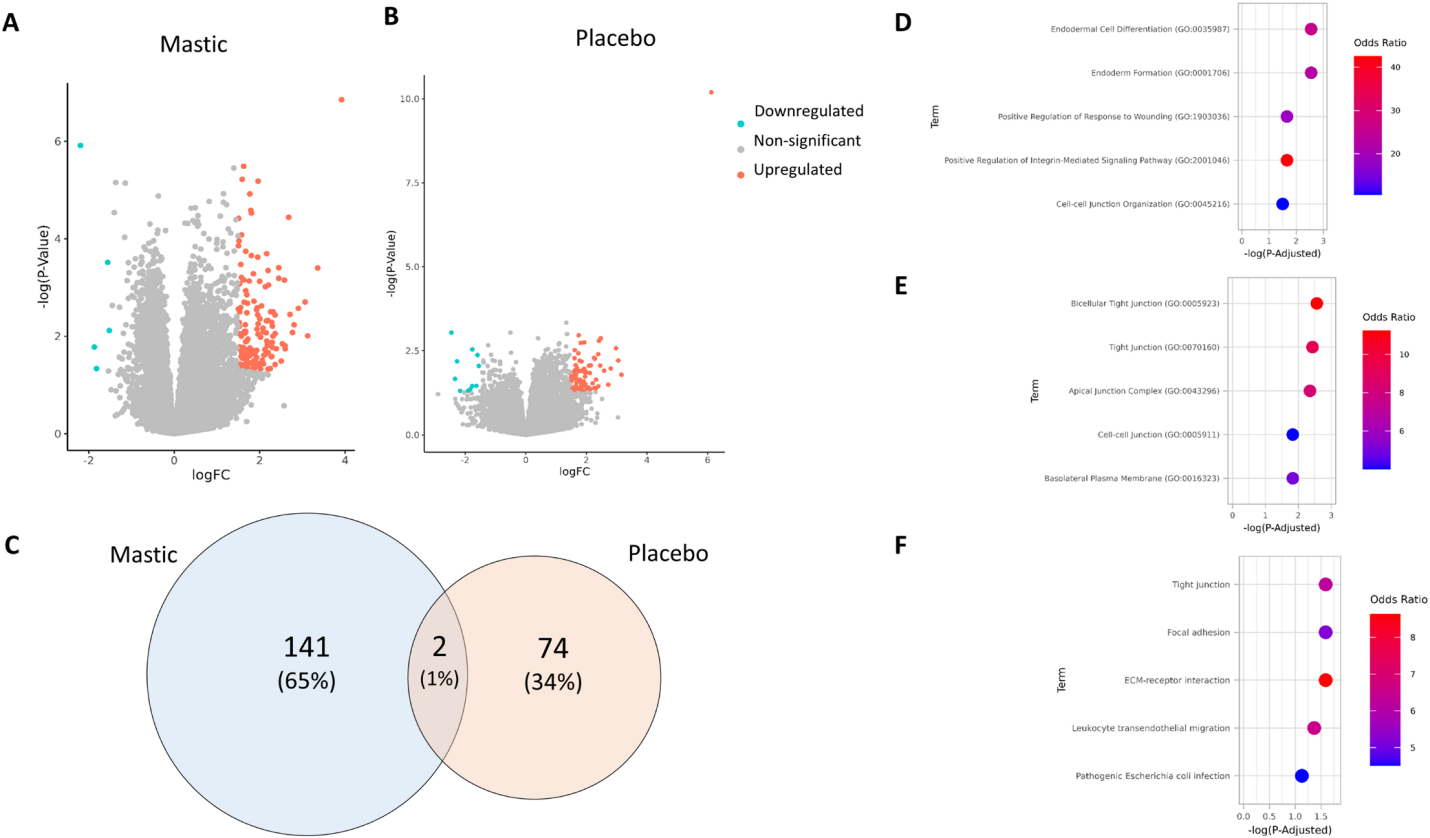
Mastic treatment leads to transcriptome profile changes in high‐risk patients with hypertension. Volcano plots depicting differentially expressed genes (DEGs) derived from comparisons regarding the (A) mastic and (B) placebo treatments compared with the condition before the respective treatment. Cut‐offs for volcano plots: *p*‐value < 0.05, log_2_ fold‐change > 1.5 and < −1.5. (C) Venn diagram depicting the number of common and exclusive upregulated DEGs derived from treatment with mastic or placebo. Gene Ontology—(D) biological process and (E) cellular component for the upregulated DEGs after mastic treatment. DEGs derived after placebo treatment did not generate statistically significant terms. (F) Functional enrichment analysis (KEGG) for the upregulated DEGs after mastic treatment.

## 4 Discussion

In this double‐blind, randomized, placebo‐controlled HYPER‐MASTIC trial we evaluated whether Chios mastic supplementation can reduce residual cardiovascular risk in well‐controlled hypertensive patients by examining BP, vascular function, oxidative stress and gene expression. Mastic has previously emerged as a promising natural antihypertensive strategy, with preclinical and clinical evidence of blood‐pressure‐lowering and vascular benefits [20, 21].

Our principal finding is that, in well‐controlled hypertensive individuals with low baseline cardiovascular risk, Chios mastic supplementation does not confer additional short‐term antihypertensive benefit at the tested doses, likely reflecting a ceiling effect. cSBP24hr showed a significant time‐by‐group interaction driven by an increase in the low‐dose group, a pattern that persisted in the high‐risk subgroup analysis. Importantly, adherence was high and confirmed objectively by dose‐dependent increases in plasma MNA and IMNA, so the neutral BP findings cannot be attributed to poor compliance. ROS and proteasome activity in PBMCs and RBCs were also unchanged‐contrasting with earlier acute and experimental studies [19, 32]—likely reflecting the longer exposure, the well‐controlled clinical state of participants and their relatively low inflammatory and oxidative burden.

Previous results from MAST4HEALTH [33, 34] consistently showed mastic‐related improvements in metabolic, inflammatory and oxidative‐stress markers, with stronger effects in patients with higher cardiometabolic or inflammatory burden, whereas BP effects were modest or not primary outcomes. This is consistent with the present results, where antihypertensive effects were absent in well‐controlled patients yet mastic‐specific molecular and vascular transcriptomic changes emerged in the high‐risk subgroup. Similarly, Gioxari et al. [21] reported that Chios mastiha essential oil for 3 months in metabolically unhealthy adults improved systolic BP, lipids, adiposity and oxidized LDL. Together, these data support the notion that mastic‐related interventions exert clearer cardiometabolic benefits in populations with greater baseline metabolic and inflammatory burden. Other nutraceuticals such as quercetin and resveratrol show similar patterns, with effects driven by baseline BP and risk and smaller average reductions in lower‐risk populations [35], consistent with a ceiling effect in well‐managed patients.

Some baseline imbalances should be considered when interpreting these results. The low‐dose group was significantly younger and more frequently reported a family history of hypertension, although other characteristics were comparable; analyses were adjusted for age and sex. Younger age and inherited susceptibility may influence vascular adaptability, possibly contributing to the greater short‐term variability seen in this group. Together with regression to the mean and placebo responsiveness, these features likely contributed to response heterogeneity rather than indicating any detrimental effect of Chios mastic.

In the high‐risk subgroup‐characterized by higher inflammatory burden or advanced age‐mastic supplementation induced distinct gene‐expression changes compared with placebo, particularly in pathways of tight junction organization, cell–cell adhesion and endothelial integrity. These changes suggest that Chios mastic may influence vascular homeostasis and endothelial biology even in the absence of short‐term BP reduction. The absence of comparable transcriptomic changes in the placebo group supports a mastic‐specific biological effect. Complementary analyses from the HYPER‐MASTIC project also show modulation of microRNA‐21—an epigenetic biomarker of hypertension and vascular remodeling‐particularly in night‐time BP parameters [12], reinforcing a biologically meaningful effect of mastic at the molecular level.

These findings should be interpreted in light of certain limitations. A larger sample would enhance representativeness and robustness. The 3‐month duration may be insufficient to capture longer‐term vascular and clinical benefits, particularly for nutraceutical interventions whose effects may emerge gradually. Because cardiovascular risk factors were already well controlled at enrolment, pro‐hypertensive mechanisms were likely attenuated, limiting the ability to detect additive effects. These limitations are partly offset by the rigorous randomized controlled design and the team's experience with previous clinical interventions.

In conclusion, Chios mastic supplementation did not lower BP in well‐controlled hypertensive patients over 3 months, but it appears to exert biologically meaningful effects on vascular‐related gene expression, particularly in individuals with higher residual cardiovascular risk. Future studies with longer follow‐up, higher‐risk populations and integrated molecular and clinical endpoints are warranted to clarify the role of Chios mastic as an adjunctive cardiovascular intervention.

## Author Contributions

**Ioanna Panagiota Kalafati:** investigation, formal analysis, writing – review and editing, writing – original draft, data curation. **Anastasia‐Areti Kyriazopoulou‐Korovesi:** investigation, writing – review and editing. **Evangelia Mentsiou‐Nikolaou:** investigation, writing – original draft, writing – review and editing. **Maria Papaioannou:** investigation, writing – review and editing. **Christina Konstantaki:** writing – review and editing, investigation. **Lasthenis Angelidakis:** investigation, writing – review and editing. **Despoina D. Gianniou:** investigation, writing – review and editing. **Eleni V. Mikropoulou:** investigation, writing – review and editing. **Panagiota Giardoglou:** writing – review and editing, investigation. **Dimitrios Valakos:** formal analysis, writing – review and editing. **Giannis Vatsellas:** conceptualization, investigation, writing – review and editing. **HYPER‐MASTIC Consortium:** writing – review and editing. **Ioanna Andreadou:** writing – review and editing, methodology. **Efstathios Manios:** investigation, writing – review and editing. **Maria Halabalaki:** methodology, writing – review and editing. **Ioannis P. Trougakos:** methodology, writing – review and editing. **George V. Dedoussis:** methodology, writing – review and editing. **Kimon Stamatelopoulos:** conceptualization, methodology, investigation, writing – review and editing, supervision.

## Funding

This research was co‐financed by the European Regional Development Fund of the European Union and Greek national funds through the Operational Program Competitiveness, Entrepreneurship and Innovation, under the call RESEARCH–CREATE–INNOVATE (project code: T2EDK‐00547). The funders had no role in the study design, data collection and analysis, decision to publish, or preparation of the manuscript.

## Ethics Statement

The HYPER‐MASTIC trial was conducted in accordance with the principles of the Declaration of Helsinki and the ICH Good Clinical Practice guidelines. The study protocol was approved by the Ethics Committee of Harokopio University of Athens (approval number 35‐21/12/2021) and by the Scientific Board of “Alexandra” General Hospital (approval number 8/23‐12‐2021). Written informed consent was obtained from all participants prior to enrollment.

## Conflicts of Interest

The authors declare no conflicts of interest.

## Supporting information


**Figure S1:** Plasma MNA + IMNA levels among the three groups during the intervention. Box plot showing the change in plasma MNA + IMNA concentrations from baseline to 3 months across the three intervention groups (placebo, low‐dose, high‐dose). Levels remained unchanged in placebo, increased in the low‐dose group, and rose approximately three‐fold further in the high‐dose group, confirming objective adherence to the intervention. IMNA, isomasticadienonic acid; MNA, masticadienonic acid.
**Table S1:** MNA + IMNA levels of the three groups during the intervention. Values are presented as mean ± SD, median (interquartile range; IQR), or relative frequencies as appropriate. *p*‐values calculated with ANOVA or Kruskal–Wallis. IMNA, isomasticadienonic acid; MNA, masticadienonic acid.
**Figure S2:** Mean difference of total SBP measurements (ΔSBP) over the 3‐month intervention across the three groups. Comparisons among the three groups were performed using one‐way ANOVA, whereas between‐group differences for all pairwise combinations were assessed using the Bonferroni post hoc test. ΔSBP: Mean difference of total systolic blood pressure over the three‐month intervention.
**Figure S3:** Mean difference of total DBP measurements (ΔDBP) over the 3‐month intervention across the three groups. Comparisons among the three groups were performed using one‐way ANOVA, whereas between‐group differences for all pairwise combinations were assessed using the Bonferroni post hoc test. ΔDBP: Mean difference of total diastolic blood pressure over the three‐month intervention.
**Table S2:** Daytime measurements of 24‐h Holter monitoring among groups. The *p*‐value for time was calculated using paired‐sample *t*‐tests to compare pre‐ and post‐intervention levels within each treatment group. Between‐group differences at each time point (P groups) were assessed using one‐way ANOVA, and the time × group interaction was evaluated using repeated‐measures ANOVA, adjusted for age and sex. Significant values shown in bold italic. cSBP/cDBP, central systolic/diastolic blood pressure; DBP, diastolic blood pressure; PWV, pulse wave velocity; SBP, systolic blood pressure.
**Table S3:** Night‐time measurements of 24‐h Holter monitoring among groups. Statistical methods as in Table S2. cSBP/cDBP, central systolic/diastolic blood pressure; DBP, diastolic blood pressure; PWV, pulse wave velocity; SBP, systolic blood pressure.
**Table S4:** BMI and blood biochemical measurements: changes from baseline to final visit. Statistical methods as in Table S2. BMI, body mass index; CRP, C‐reactive protein; Glu, glucose; HDL, high‐density lipoprotein cholesterol; LDL, low‐density lipoprotein cholesterol; TC, total cholesterol; TG, triglycerides.
**Table S5:** Proteasome and ROS levels in PBMCs and RBCs over the 3‐month intervention. Statistical methods as in Table S2. Abs, absolute value; Fluo%, fluorescence percentage relative to baseline (set at 100%); PBMCs, peripheral blood mononuclear cells; RBCs, red blood cells; ROS, reactive oxygen species.
**Table S6:** Baseline characteristics of high‐risk participants by treatment group (*n* = 70). Values are presented as mean ± SD for normally distributed variables, as median (interquartile range; IQR) for non‐normally distributed variables, or as relative frequencies for categorical variables. *p*‐values were calculated with one‐way ANOVA or Kruskal–Wallis tests according to the normality of distribution. *Significant pairwise differences based on Bonferroni post hoc test. AH, arterial hypertension; CAD, coronary artery disease; DM2, diabetes mellitus type 2; MET, metabolic equivalent.
**Table S7:** Total 24‐h Holter measurements pre‐ and post‐intervention in the high‐risk subgroup. Statistical methods as in Table S2. cSBP/cDBP, central systolic/diastolic blood pressure; DBP, diastolic blood pressure; PWV, pulse wave velocity; SBP, systolic blood pressure.
**Table S8:** Office blood pressure and vascular measurements in the high‐risk subgroup. Statistical methods as in Table S2. cSBP/cDBP, central systolic/diastolic blood pressure; DBP, diastolic blood pressure; FMD, flow‐mediated dilatation; PWV, pulse wave velocity; SBP, systolic blood pressure.
**Table S9:** BMI and blood biochemical measurements in the high‐risk subgroup. Statistical methods as in Table S2. BMI, body mass index; CRP, C‐reactive protein; Glu, glucose; HDL, high‐density lipoprotein cholesterol; LDL, low‐density lipoprotein cholesterol; TC, total cholesterol; TG, triglycerides.
**Table S10:** Baseline characteristics of non‐CAD participants by treatment group (*n* = 72). Statistical methods as in Table S6. CAD, coronary artery disease; DM2, diabetes mellitus type 2; MET, metabolic equivalent.
**Table S11:** Total 24‐h Holter monitoring pre‐ and post‐intervention in non‐CAD participants. Statistical methods as in Table S2. cSBP/cDBP, central systolic/diastolic blood pressure; DBP, diastolic blood pressure; PWV, pulse wave velocity; SBP, systolic blood pressure.
**Table S12:** Office blood pressure and vascular measurements in non‐CAD participants. Statistical methods as in Table S2. cSBP/cDBP, central systolic/diastolic blood pressure; DBP, diastolic blood pressure; FMD, flow‐mediated dilatation; PWV, pulse wave velocity; SBP, systolic blood pressure.
**Table S13:** BMI and blood biochemical measurements in non‐CAD participants. Statistical methods as in Table S2. BMI, body mass index; CRP, C‐reactive protein; Glu, glucose; HDL, high‐density lipoprotein cholesterol; LDL, low‐density lipoprotein cholesterol; TC, total cholesterol; TG, triglycerides.

## Data Availability

De‐identified individual participant data and the study protocol will be made available to qualified researchers with a methodologically sound proposal, after approval by the HYPER‐MASTIC steering committee and a signed data‐sharing agreement. Requests should be directed to the corresponding author. Raw RNA‐sequencing data will be deposited in the Gene Expression Omnibus (GEO) repository upon publication; the accession number will be provided at that stage.
