# Supplementary material for: Chios Mastic in Well‐Controlled Hypertension: Findings From the HYPER‐MASTIC Randomized Controlled Trial
Source: Pharmacol Res Perspect. 2026 Oct 15;14(6):e70322. doi: 10.1002/prp2.70322 (PMC13673137; doi:10.1002/prp2.70322)
Supplement: Supplementary file 1 [file PRP2-14-e70322-s001.pdf]

Supplementary Material

Supplementary Methods

Blood sampling and isolation of PBMCs and RBCs

After blood drainage, whole blood was diluted with phosphate-buffered saline (PBS) (1:1) to enable PBMC and RBC isolation. The diluted whole blood was then carefully layered over the separation medium, Pancoll [density 1.077 g/mL (PAN BIOTECH)] (at half the sample volume), ensuring that the two phases were kept separated before the centrifugation. Tubes were centrifuged at 400 × g for 30 min at 20 °C (acceleration 9, braking rate 0). PBMCs were collected after aspiration of the plasma and platelet layer. For RBC isolation, the upper layer of polymorphonuclear leukocytes and a portion of the RBC phase were also aspirated, leaving the RBC sediment undisturbed; the highly enriched RBC fraction was then collected. Both cell types were washed with PBS and processed for downstream assays. Stored buffy coat samples were further used for DNA extraction (NucleoSpin Tissue, Macherey-Nagel) and stored at −80 °C for genotyping.

ROS measurement

To determine ROS levels, collected cells in PBS were incubated with CM-H2DCFDA dye (Invitrogen, Carlsbad, CA, USA) for 30 min at 37 °C in the dark. Following centrifugation and dye removal, cells were lysed in Nonidet P-40 lysis buffer (1% Nonidet P-40, 150 mM NaCl, 50 mM Tris, pH 8.0) and cleared by centrifugation at 19,000 × g for 10 min at 4 °C. Protein content was adjusted using the Bradford assay (Bio-Rad, Hercules, CA, USA). Fluorescent dichlorodihydrofluorescein, used to detect ROS, was measured on a Spark® Tecan microplate reader (Tecan Group Ltd., Männedorf, Switzerland) at excitation 490 nm/emission 520 nm.

Proteasome activity measurement

To measure proteasome peptidase activity, cells were lysed on ice in a buffer suitable for the isolation of intact 26S proteasomes (0.2% NP-40, 5 mM ATP [fresh], 10% glycerol, 20 mM KCl, 1 mM EDTA, 1 mM DTT, 20 mM Tris, pH 7.6). Lysates were cleared by centrifugation at 19,000 × g (4 °C) and protein content adjusted with Bradford. Cleared supernatants were used immediately to determine the chymotrypsin-like (CT-L/LLVY) proteasome activity by recording (excitation 350 nm; emission 440 nm) the hydrolysis of the fluorogenic peptide Suc-Leu-Leu-Val-Tyr-AMC (Enzo Life Sciences, Farmingdale, NY, USA) at 37 °C for 30 min on a Spark® Tecan microplate reader.

Quantification of plasma MNA and IMNA

Masticadienonic acid (MNA) and isomasticadienonic acid (IMNA) levels in participants' plasma were measured as previously described (1,2) with slight modifications. Plasma samples were extracted by protein precipitation with ice-cold acetonitrile, evaporated to dryness, and reconstituted in 100 μL of mobile phase containing 5 ng/mL of the internal standard (acetyl ursolic acid, AUA). Analyses were performed on an Acquity H-class UPLC system (Waters, Milford, MA, USA) hyphenated to an AB Sciex (Marlborough, MA, USA) TripleTOF 5600+ mass spectrometer equipped with a DuoSpray ion source. Data acquisition was performed using Analyst 1.7.1 software (AB Sciex), and spectral interpretation and analyte quantification with Sciex OS (version 1.7, AB Sciex). Separation was achieved on an Acquity HSS T3 (Waters) column (100 × 2.1 mm, 1.8 μm) using a gradient of water containing 0.1% (v/v) formic acid and acetonitrile.

RNA sequencing analysis

RNA-seq experiments were carried out in the Greek Genome Center (GGC) of the Biomedical Research Foundation of the Academy of Athens (BRFAA). RNA-seq libraries were prepared with the NEBNext Ultra II Directional RNA Library Prep Kit for Illumina, with 1 μg of total RNA input. Library QC was performed with the Agilent Bioanalyzer DNA1000 kit and quantitation with the Qubit HS spectrophotometric method. At least 15 million 101-bp single-end reads were generated per sample. Quality control of the FASTQ raw data was performed using FASTQC. FASTQ files were aligned to the human genome (hg19) using HISAT2 (3). Counts were defined using htseq-count (HTSeq v2.0.3) (4) in "intersection non-empty" mode, with the reverse strandness option, after removing the haemoglobin gene annotations from the hg19 GTF file. Count files were used as input for DESeq2 (5). Normalization was performed with the estimateSizeFactors function followed by Differentially Expressed Genes (DEGs) analysis. DEGs were classified as those with log2 fold-change <-1.5 or >1.5 and p-value ≤ 0.05. Functional enrichment analysis was performed using the EnrichR web tool (6).

Supplementary Results

Supplementary Figure 1. Plasma MNA + IMNA levels among the three groups during the intervention.

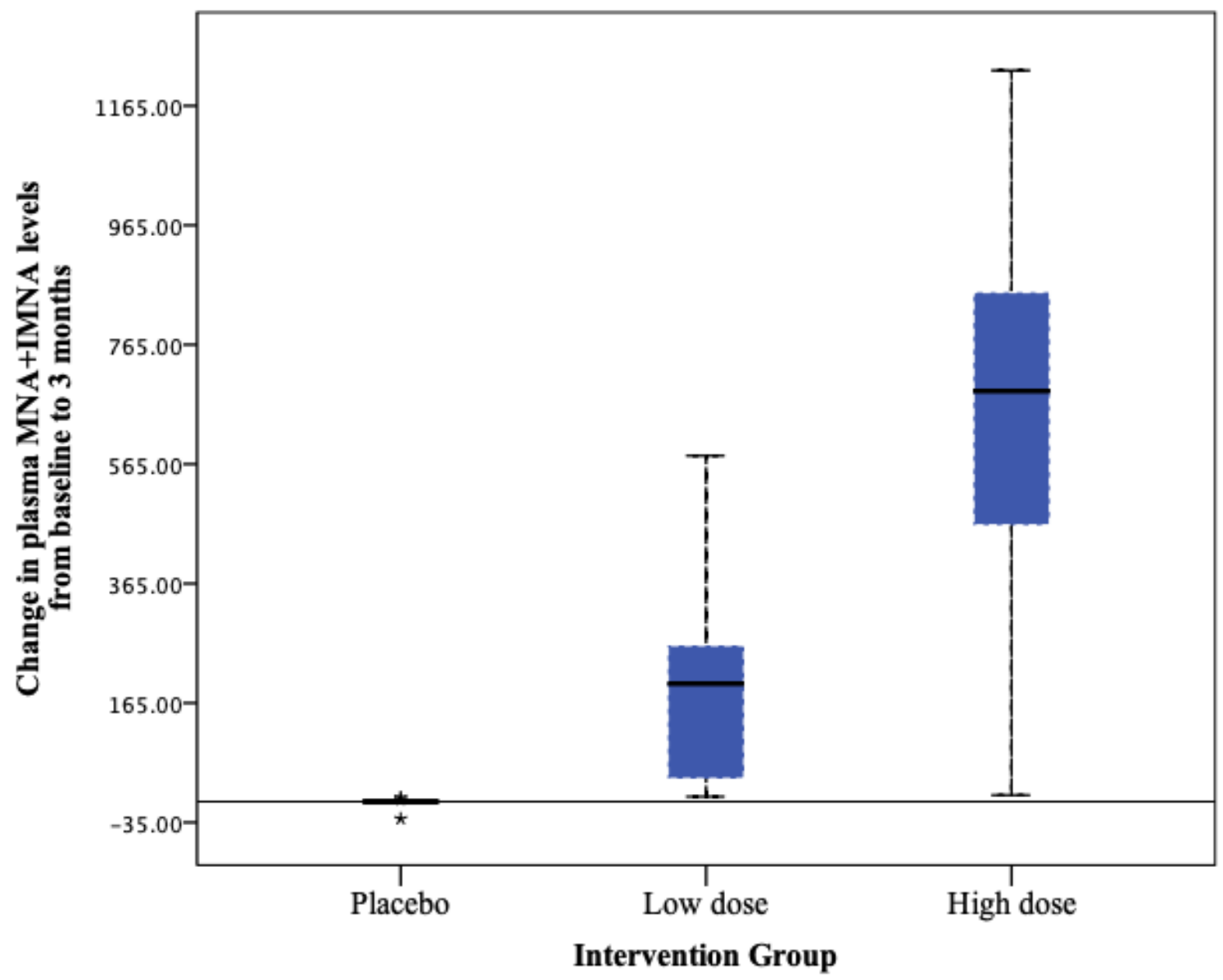

Box plot showing the change in plasma MNA + IMNA concentrations from baseline to 3 months across the three intervention groups (placebo, low-dose, high-dose). Levels remained unchanged in placebo, increased in the low-dose group, and rose approximately three-fold further in the high-dose group, confirming objective adherence to the intervention. MNA; Masticadienonic acid, IMNA; Isomasticadienonic acid.

Supplementary Table 1. MNA + IMNA levels of the three groups during the intervention.

Variable	Placebo (N=14)	Low dose (N=9)	High dose (N=8)	p-value	P time*group
MNA+IMNA baseline	0 (0)	0 (6.9)	0 (0)	0.82	***<0.001***
MNA+IMNA follow-up	0 (0.59)*	197.3 (295.14)*	687.35 (443.68)*	***<0.001***	

Values are presented as mean ± SD, median (interquartile range; IQR), or relative frequencies as appropriate. P-values calculated with ANOVA or Kruskal–Wallis. MNA; Masticadienonic acid, IMNA; Isomasticadienonic acid.

Supplementary Figure 2. Mean difference of total SBP measurements (ΔSBP) over the 3-month intervention across the three groups.

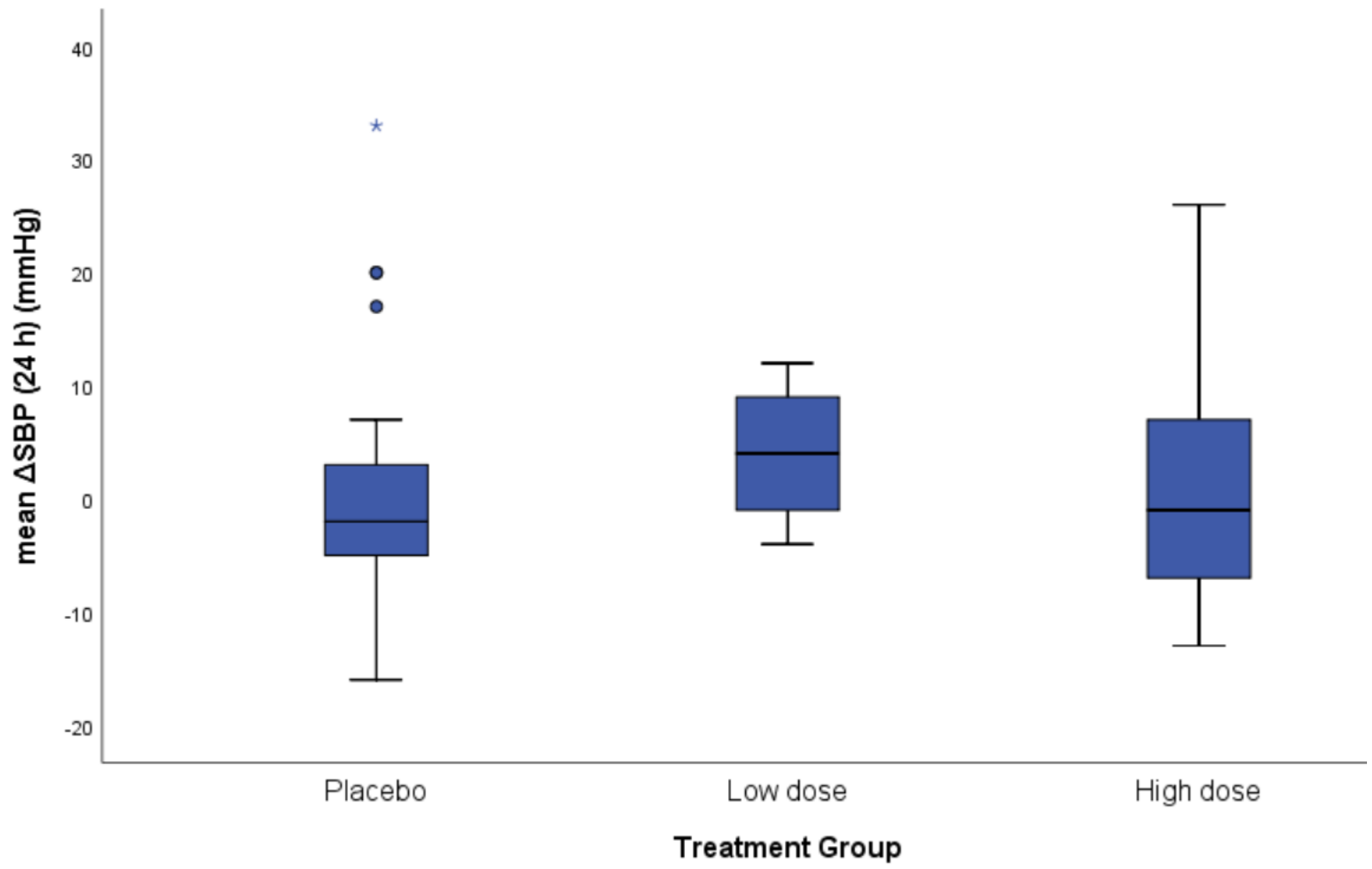

Comparisons among the three groups were performed using one-way ANOVA, while between-group differences for all pairwise combinations were assessed using the Bonferroni post-hoc test. ΔSBP: Mean difference of total Systolic Blood Pressure over the three-month intervention.

Supplementary Figure 3. Mean difference of total DBP measurements (ΔDBP) over the 3-month intervention across the three groups.

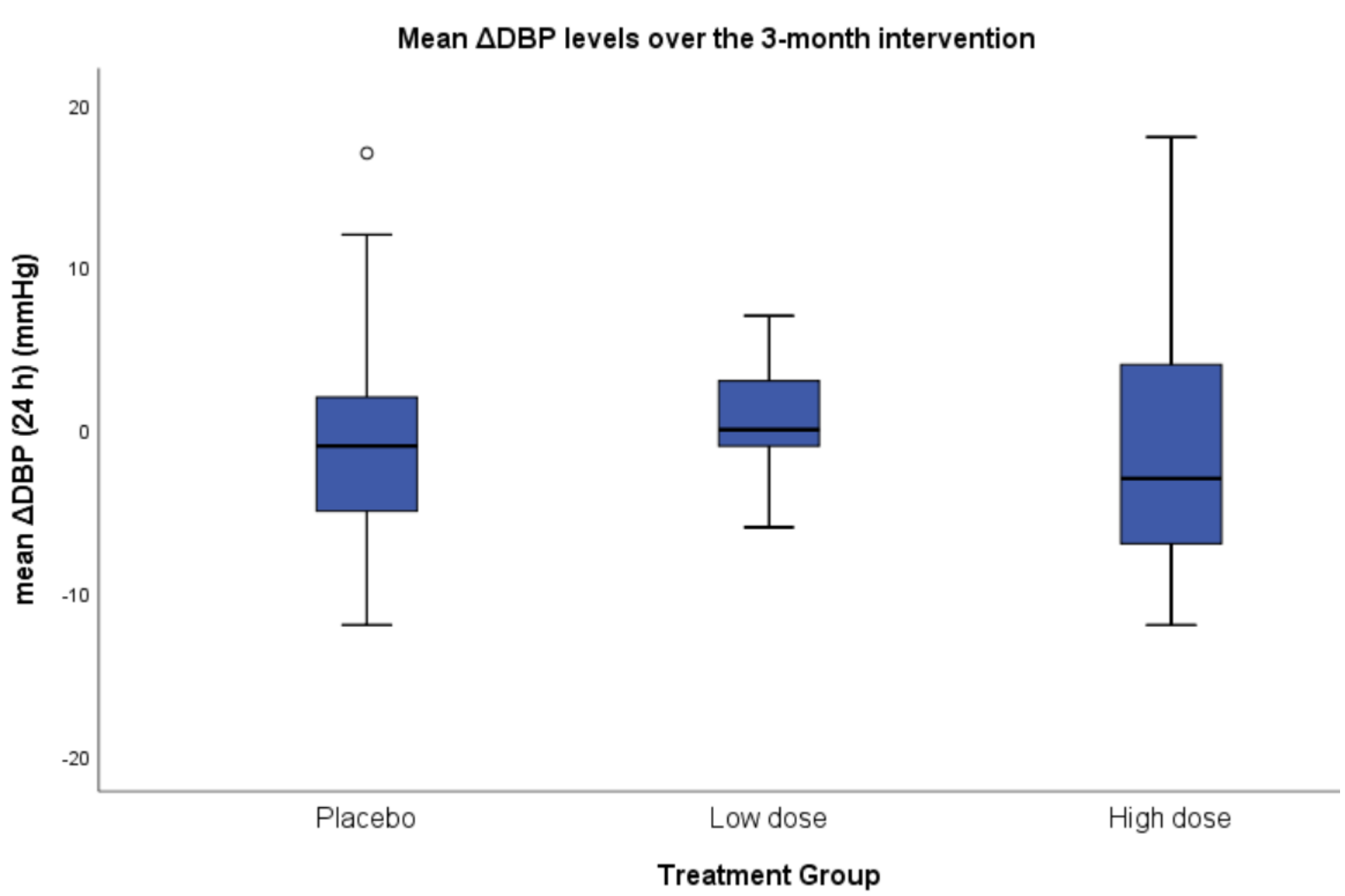

Comparisons among the three groups were performed using one-way ANOVA, while between-group differences for all pairwise combinations were assessed using the Bonferroni post-hoc test. ΔDBP: Mean difference of total Diastolic Blood Pressure over the three-month intervention.

Supplementary Table 2. Daytime measurements of 24-h Holter monitoring among groups.

Variable	Time	Placebo (N=31)	Low dose (N=25)	High dose (N=25)	P groups	P time*group
SBPday (mmHg)	Baseline	123.74 ± 9.50	125.04 ± 7.34	124.96 ± 7.58	0.74	0.43
	3rd month	124.87 ± 9.04	128.4 ± 7.75	126.58 ± 8.96	0.32	
	P time	0.57	***0.005***	0.41		
DBPday (mmHg)	Baseline	77.65 ± 8.97	78.28 ± 7.66	79.13 ± 8.45	0.82	0.23
	3rd month	77.13 ± 8.44	79.68 ± 7.86	78.46 ± 8.18	0.51	
	P time	0.66	0.09	0.67		
PWVday (m/s)	Baseline	8.89 ± 1.03	8.22 ± 0.91*	8.90 ± 1.33*	***0.032***	0.49
	3rd month	8.94 ± 0.94	8.38 ± 1.02	9.17 ± 1.50	0.05	
	P time	0.40	***0.002***	0.20		
cSBPday (mmHg)	Baseline	124.81 ± 9.57	123.04 ± 8.39	127.46 ± 8.42	0.43	0.24
	3rd month	126.13 ± 9.20	129.84 ± 8.57	128.75 ± 12.30	0.25	
	P time	0.52	***0.041***	0.55		
cDBPday (mmHg)	Baseline	78.97 ± 9.19	79.88 ± 7.42	80.83 ± 8.57	0.74	0.08
	3rd month	78.74 ± 8.75	82.08 ± 7.54	79.75 ± 8.05	0.31	
	P time	0.87	***0.015***	0.49		

The p-value for time was calculated using paired-sample t-tests to compare pre- and post-intervention levels within each treatment group. Between-group differences at each time point (P groups) were assessed using one-way ANOVA, and the time×group interaction was evaluated using repeated-measures ANOVA, adjusted for age and sex. Significant values shown in bold italic. SBP, systolic blood pressure; DBP, diastolic blood pressure; PWV, pulse wave velocity; cSBP/cDBP, central systolic/diastolic blood pressure.

Supplementary Table 3. Night-time measurements of 24-h Holter monitoring among groups.

Variable	Time	Placebo (N=31)	Low dose (N=25)	High dose (N=25)	P groups	P time*group
SBPnight (mmHg)	Baseline	114.97 ± 11.87	115.39 ± 10.35	116.38 ± 12.06	0.80	0.11
	3rd month	115.10 ± 12.61	119.91 ± 9.14	116.46 ± 8.55	0.29	
	P time	0.96	***0.014***	0.97		
DBPnight (mmHg)	Baseline	69.62 ± 9.81	70.74 ± 9.64	70.54 ± 11.76	0.89	0.52
	3rd month	69.14 ± 8.55	70.96 ± 8.16	69.58 ± 9.85	0.74	
	P time	0.78	0.90	0.62		
PWVnight (m/s)	Baseline	8.61 ± 1.13	7.95 ± 1.00*	8.64 ± 1.28*	***0.03***	0.60
	3rd month	8.66 ± 1.12	8.12 ± 1.05	8.88 ± 1.48	0.08	
	P time	0.58	***0.007***	0.29		
cSBPnight (mmHg)	Baseline	123.52 ± 12.23	122.91 ± 8.56	125.92 ± 14.15	0.22	0.17
	3rd month	124.97 ± 13.46	127.73 ± 8.40	126.08 ± 13.68	0.76	

Variable	Time	Placebo (N=31)	Low dose (N=25)	High dose (N=25)	P groups	P time*group
	P time	0.58	***0.014***	0.94		
cDBPnight (mmHg)	Baseline	70.55 ± 9.72	73.36 ± 9.38	72.21 ± 12.09	0.88	0.95
	3rd month	71.62 ± 8.44	72.32 ± 8.48	72.00 ± 9.61	0.96	
	P time	0.57	0.59	0.91		

Statistical methods as in Supplementary Table 2. SBP, systolic blood pressure; DBP, diastolic blood pressure; PWV, pulse wave velocity; cSBP/cDBP, central systolic/diastolic blood pressure.

Supplementary Table 4. BMI and blood biochemical measurements: changes from baseline to final visit.

Variable	Time	Placebo (N=31)	Low dose (N=25)	High dose (N=25)	P groups	P time*group
BMI (kg/m²)	Baseline	27.34 (5.8)	29.36 (6.1)	28.41 (5.7)	0.24	0.24
	3rd month	27.97 (4.8)	28.74 (6.6)	27.43 (5)	0.44	
	P time	0.72	0.58	0.29		
Glu (mg/dL)	Baseline	94 (15)	93 (13)	95 (14)	0.83	***0.017***
	3rd month	93.5 (12)	95.5 (19)	95.5 (14)	0.33	
	P time	***0.05***	0.28	0.18		
TC (mg/dL)	Baseline	158 (48)	162 (55)	163.5 (77)	0.95	0.62
	3rd month	155 (56)	158 (61)	170 (59)	0.74	
	P time	0.49	0.27	***0.04***		
TG (mg/dL)	Baseline	99 (52)	102 (59)	77 (48)	0.19	0.35
	3rd month	105 (44)	112.5 (53)	80 (54)	0.054	
	P time	0.62	***0.05***	0.74		
HDL (mg/dL)	Baseline	57.58 ± 12.25	49.08 ± 12.61	54.6 ± 15.04	0.06	0.57
	3rd month	56.94 ± 11.55*	47.92 ± 13.24*	53.96 ± 14.35	***0.02***	
	P time	0.69	0.60	0.69		
LDL (mg/dL)	Baseline	78 (38)	94 (55)	85 (52)	0.54	0.09
	3rd month	90.52 ± 34.75	100.75 ± 33.71	103.12 ± 41.43	0.37	
	P time	0.36	0.45	***0.03***		
CRP (mg/L)	Baseline	1.28 (1.94)	1.66 (3.71)	0.95 (2.19)	0.38	0.62
	3rd month	0.91 (2.61)	1.62 (2.61)	1.23 (1.28)	0.36	
	P time	0.62	0.64	0.98		

Statistical methods as in Supplementary Table 2. BMI, body mass index; Glu, glucose; TC, total cholesterol; TG, triglycerides; HDL, high-density lipoprotein cholesterol; LDL, low-density lipoprotein cholesterol; CRP, C-reactive protein.

Supplementary Table 5. Proteasome and ROS levels in PBMCs and RBCs over the 3-month intervention.

Variable	Time	Placebo (N=27)	Low dose (N=24)	High dose (N=21)	P groups	P time*group
Proteasome PBMCs (Abs/μg)	Baseline	3170.98 ± 1859.91	2756.01 ± 2068.73	3930.90 ± 2128.22	0.27	0.55
	3rd month	3470.62 (2817.5)	3347.55 (2181.31)	3470.2 (2368.94)	0.79	
	P time	0.82	0.27	0.53		
Proteasome PBMCs (Fluo%)	Baseline	100 (0)	100 (0)	100 (0)	1	0.66
	3rd month	98.38 (98.15)	142.12 (348.10)	104.75 (85.82)	0.14	
	P time	0.17	***0.019***	0.19		
Proteasome RBCs (Abs/μg)	Baseline	202.06 (202.11)	197.3 (232.61)	300.27 (402.11)	0.11	0.56
	3rd month	180.59 (183.78)	196.33 (180.41)	226.35 (311.72)	0.62	
	P time	0.94	0.77	0.71		
Proteasome RBCs (Fluo%)	Baseline	100 (0)	100 (0)	100 (0)	1	0.25
	3rd month	101.43 (127.38)	109.03 (273.9)	78.23 (108.21)	0.19	
	P time	0.07	***0.026***	0.76		
ROS PBMCs (Abs/μg)	Baseline	2437.13 ± 2014.58	2275.80 ± 1344.36	2139.59 ± 1272.39	0.70	0.82
	3rd month	1738.04 ± 1085.66	2254.77 ± 1324.52	2424.76 ± 1518.39	0.19	
	P time	0.08	0.95	0.40		
ROS PBMCs (Fluo%)	Baseline	100 (0)	100 (0)	100 (0)	1	0.46
	3rd month	82.78 (93.11)	98.53 (186.45)	105.98 (218.13)	0.88	
	P time	0.29	0.11	***0.03***		
ROS RBCs (Abs/μg)	Baseline	2118.03 (2813.72)	1069 (1675.23)	1552.75 (2767.33)	0.46	0.33
	3rd month	1314.23 (1506.8)	1212.58 (2528.59)	1087.10 (952.75)	0.64	
	P time	0.07	0.85	0.13		
ROS RBCs (Fluo%)	Baseline	100 (0)	100 (0)	100 (0)	0.22	0.82
	3rd month	96.92 (201.93)	126.44 (260.41)	101.37 (131.89)	0.71	
	P time	0.12	0.14	0.87		

Statistical methods as in Supplementary Table 2. PBMCs, peripheral blood mononuclear cells; RBCs, red blood cells; ROS, reactive oxygen species; Abs, absolute value; Fluo%, fluorescence percentage relative to baseline (set at 100%).

Analyses in the high-risk subgroup

Supplementary Table 6. Baseline characteristics of high-risk participants by treatment group (n=70).

Variable	Placebo (N=26)	Low dose (N=15)	High dose (N=19)	p-value
Age (years)	64.92 ± 5.88	60.13 ± 6.94	63.79 ± 9.7	0.29
Female sex (%)	57.7	46.7	52.6	0.79
BMI (kg/m²)	28.64 ± 4.56	29.71 ± 4.24	30.12 ± 4.64	0.54
Years since diagnosis of AH	10 (19)	8 (12)	14 (13)	0.64
Family history of AH (% Yes)	50	80	84.2	***0.027***
Family history of CAD (% Yes)	26.9	53.3	31.6	0.22
Family history of hyperlipidemia (% Yes)	50	80	84.2	0.027
DM2 (% Yes)	26.9	26.7	21.1	0.89
CAD (% Yes)	11.5	13.3	5.3	0.70
Hyperlipidemia (% Yes)	69.2	60	68.4	0.82
MET min per week	1604 (1772.5)	947 (754)	1172 (1180)	0.22
Smokers (%)	34.6	26.7	21.1	0.69
Pack-Years	10 (23)	15 (25)	0.5 (27)	0.59
Energy intake (kcal)	1547.34 ± 565.95	1343.88 ± 243.49	1259.62 ± 505.15	0.43

*Values are presented as mean ± SD for normally distributed variables, as median (interquartile range; IQR) for non-normally distributed variables, or as relative frequencies for categorical variables. P-values were calculated with one-way ANOVA or Kruskal-Wallis tests according to the normality of distribution. *Significant pairwise differences based on Bonferroni post-hoc test. AH, arterial hypertension; CAD, coronary artery disease; DM2, diabetes mellitus type 2; MET, metabolic equivalent.*

Supplementary Table 7. Total 24-hour Holter measurements pre- and post-intervention in the high-risk subgroup.

Variable	Time	Placebo (N=26)	Low dose (N=15)	High dose (N=19)	P groups	P time*group
SBP24hr (mmHg)	Baseline	123.27 ± 9.45	123.8 ± 9.02	123.84 ± 8.41	0.97	0.18
	3rd month	123.27 ± 8.78	126.73 ± 9.09	122.33 ± 7.01	0.31	
	P time	1.00	***0.031***	0.54		
DBP24hr (mmHg)	Baseline	76.31 ± 9.35	75.67 ± 8.63	77.58 ± 8.56	0.81	0.20
	3rd month	74.81 ± 7.08	76.13 ± 8.85	74.94 ± 7.50	0.86	
	P time	0.24	0.61	0.11		
PWV24hr (m/s)	Baseline	9.03 ± 0.98	8.47 ± 0.88	9.10 ± 1.40	0.21	0.28
	3rd month	8.9 (1.2)	8.6 (1.6)	9.5 (2.8)	0.38	
	P time	0.71	***0.034***	0.79		
cSBP24hr (mmHg)	Baseline	124.5 (14)	125 (14)	130 (16)	0.68	***0.024***
	3rd month	126.23 ± 9.27	130.07 ± 9.07	126.56 ± 8.03	0.30	
	P time	0.69	***0.022***	0.42		

Variable	Time	Placebo (N=26)	Low dose (N=15)	High dose (N=19)	P groups	P time*group
cDBP24hr (mmHg)	Baseline	77.38 ± 9.5	76.73 ± 8.33	78.68 ± 9.06	0.81	0.16
	3rd month	76.69 ± 7.31	78.6 ± 8.39	76.72 ± 7.24	0.71	
	P time	0.65	0.13	0.21		

Statistical methods as in Supplementary Table 2. SBP, systolic blood pressure; DBP, diastolic blood pressure; PWV, pulse wave velocity; cSBP/cDBP, central systolic/diastolic blood pressure.

Supplementary Table 8. Office blood pressure and vascular measurements in the high-risk subgroup.

Variable	Time	Placebo (N=26)	Low dose (N=15)	High dose (N=19)	P groups	P time*group
SBPoffice (mmHg)	Baseline	122.57 ± 12.87	127.6 ± 10.59	127.32 ± 11.05	0.31	0.68
	3rd month	121.35 ± 10.31	128.36 ± 12.43	125.79 ± 9.39	0.17	
	P time	0.68	0.48	0.75		
DBPoffice (mmHg)	Baseline	74.83 ± 8.87	77.67 ± 6.75	80.11 ± 9.28	0.14	0.78
	3rd month	73.48 ± 7.88	77.50 ± 9.71	76.50 ± 8.77	0.36	
	P time	0.58	0.83	0.66		
FMD (%)	Baseline	2.99 (3.13)	2.26 (2.83)	2.48 (1.97)	0.35	0.67
	3rd month	3.14 (3.64)	2.07 (1.19)	1.95 (2.16)	0.24	
	P time	0.76	0.98	0.55		
cSBPoffice (mmHg)	Baseline	110.5 ± 10.24	114.14 ± 12.23	111.72 ± 9.37	0.60	0.15
	3rd month	109.8 ± 9.42	115.87 ± 13.78	114.47 ± 14.67	0.29	
	P time	0.67	0.47	0.57		
cDBPoffice (mmHg)	Baseline	75.95 ± 7.18	75.5 ± 9.19	74.78 ± 7.49	0.89	0.26
	3rd month	71.35 ± 5.59	77.2 ± 11.61	74.87 ± 8.06	0.11	
	P time	***0.023***	0.63	0.95		
PWVoffice (m/s)	Baseline	9.6 (2.85)	9.3 (1.9)	10.3 (1.13)	0.37	0.81
	3rd month	9.68 ± 1.29	9.62 ± 1.33	10.59 ± 1.37	0.09	
	P time	***0.034***	0.97	0.85		

Statistical methods as in Supplementary Table 2. FMD, flow-mediated dilatation; SBP, systolic blood pressure; DBP, diastolic blood pressure; cSBP/cDBP, central systolic/diastolic blood pressure; PWV, pulse wave velocity.

Supplementary Table 9. BMI and blood biochemical measurements in the high-risk subgroup.

Variable	Time	Placebo (N=17)	Low dose (N=11)	High dose (N=11)	P groups	P time*group
BMI (kg/m²)	Baseline	28.95 (4.7)	32.2 (7.2)	29.14 (7.8)	0.38	0.77
	3rd month	28.95 (4.5)	32.02 (7.2)	29.55 (8.9)	0.68	

Variable	Time	Placebo (N=17)	Low dose (N=11)	High dose (N=11)	P groups	P time*group
	P time	0.71	0.32	0.29		
Glu (mg/dL)	Baseline	100.29 ± 12.21	96 ± 7.81	96 ± 12.09	0.18	0.07
	3rd month	95.35 ± 14.40	93.09 ± 6.7	97.36 ± 13.03	0.72	
	P time	0.78	0.07	0.19		
TC (mg/dL)	Baseline	157 (28)	178 (38)	167 (58)	0.55	0.35
	3rd month	156 ± 33.54	182.64 ± 33.43	171.09 ± 31.09	0.67	
	P time	0.77	0.19	0.16		
TG (mg/dL)	Baseline	81 (52)	102 (63)	80 (20)	0.16	0.31
	3rd month	93.76 ± 31.34	117.55 ± 40.37	108.18 ± 76.28	0.47	
	P time	0.55	0.42	0.98		
HDL (mg/dL)	Baseline	57.47 ± 12.74	52.55 ± 10.93	57.73 ± 14.04	0.26	0.91
	3rd month	57 ± 12.77	53.09 ± 10.96	55.91 ± 15.27	0.23	
	P time	0.76	0.94	0.81		
LDL (mg/dL)	Baseline	82.47 ± 23.85	107.8 ± 25.90	97.36 ± 35.44	0.37	0.56
	3rd month	85.06 ± 30.22	110 ± 32.65	99.55 ± 28.94	0.41	
	P time	0.69	0.20	0.21		
CRP (mg/L)	Baseline	1.16 (1.84)	2.5 (4.53)	2.02 (2.47)	0.20	0.44
	3rd month	3.4 ± 6.66	9.88 ± 23.08	2.58 ± 2.54	0.25	
	P time	0.73	0.45	0.93		

Statistical methods as in Supplementary Table 2. BMI, body mass index; Glu, glucose; TC, total cholesterol; TG, triglycerides; HDL, high-density lipoprotein cholesterol; LDL, low-density lipoprotein cholesterol; CRP, C-reactive protein.

Analyses in the non-CAD subgroup

Supplementary Table 10. Baseline characteristics of non-CAD participants by treatment group (n=72).

Variable	Placebo (N=28)	Low dose (N=20)	High dose (N=24)	p-value
Age (years)	63.25 ± 6.06*	56.85 ± 6.78*	62.67 ± 9.33	***0.031***
Female sex (%)	46.4	70	62.5	0.23
BMI (kg/m²)	28.52 ± 4.36	30.13 ± 3.83	29.4 ± 4.97	0.85
Years since diagnosis of AH	10 (20)	6 (10)	10 (16)	0.57
Family history of AH (% Yes)	57.1	80	83.3	0.07
Family history of CAD (% Yes)	35.7	35	37.5	0.98
Family history of hyperlipidemia (% Yes)	57.1	80	83.3	0.07
DM2 (% Yes)	29.4	20	31.6	0.79
Hyperlipidemia (% Yes)	71.4	55	66.7	0.49

Variable	Placebo (N=28)	Low dose (N=20)	High dose (N=24)	p-value
MET min per week	2113.17 ± 1660.41	1508.44 ± 1162.91	1780.35 ± 1901.13	0.32
Smokers (%)	33.3	25	16.7	0.39
Pack-Years	10 (20)	1 (23)	2 (35)	0.76
Energy intake (kcal)	1574.44 ± 530.96	1423.43 ± 362.67	1279.43 ± 552.74	0.82

Statistical methods as in Supplementary Table 6. CAD, coronary artery disease; DM2, diabetes mellitus type 2; MET, metabolic equivalent.

Supplementary Table 11. Total 24-hour Holter monitoring pre- and post-intervention in non-CAD participants.

Variable	Time	Placebo (N=28)	Low dose (N=20)	High dose (N=24)	P groups	P time*group
SBP24hr (mmHg)	Baseline	122.25 ± 9.71	123.15 ± 8.19	123.08 ± 7.84	0.92	0.54
	3rd month	123.64 ± 8.20	126.40 ± 8.32	123.75 ± 7.26	0.44	
	P time	0.48	***0.014***	0.74		
DBP24hr (mmHg)	Baseline	75.64 ± 9.11	78.05 ± 7.80	76.79 ± 8.34	0.63	0.85
	3rd month	75.11 ± 7.83	79.10 ± 8.13	75.63 ± 7.42	0.19	
	P time	0.06	0.47	***0.042***		
PWV24hr (m/s)	Baseline	8.74 ± 0.99	7.90 ± 0.77*	8.97 ± 1.39*	***0.012***	***0.006***
	3rd month	8.82 ± 0.95	8.12 ± 0.86*	9.14 ± 1.53*	***0.018***	
	P time	0.24	***0.010***	0.22		
cSBP24hr (mmHg)	Baseline	123.5 (12)	125.5 (11)	128.5 (15)	0.42	***0.027***
	3rd month	127.57 ± 8.45	129.20 ± 7.65	128.22 ± 12.29	0.76	
	P time	0.39	0.14	0.74		
cDBP24hr (mmHg)	Baseline	76.82 ± 9.21	79.75 ± 7.66	78.33 ± 8.65	0.51	0.72
	3rd month	79.04 ± 9.16	83.40 ± 7.49	79.26 ± 7.59	0.16	
	P time	1.00	0.18	0.43		

Statistical methods as in Supplementary Table 2. SBP, systolic blood pressure; DBP, diastolic blood pressure; PWV, pulse wave velocity; cSBP/cDBP, central systolic/diastolic blood pressure.

Supplementary Table 12. Office blood pressure and vascular measurements in non-CAD participants.

Variable	Time	Placebo (N=28)	Low dose (N=20)	High dose (N=24)	P groups	P time*group
SBPoffice (mmHg)	Baseline	121.56 ± 12.07	125.95 ± 9.56	126.75 ± 10	0.19	0.81
	3rd month	122 ± 10.73	126.65 ± 12.10	125.65 ± 10.24	0.38	
	P time	0.95	0.75	0.77		
DBPoffice (mmHg)	Baseline	74.96 ± 8.93	79.80 ± 8.10	80.13 ± 8.73	0.07	0.79

Variable	Time	Placebo (N=28)	Low dose (N=20)	High dose (N=24)	P groups	P time*group
	3rd month	73.86 ± 7.67	79.20 ± 8.45	76.71 ± 8.69	0.12	
	P time	0.71	0.61	0.37		
FMD (%)	Baseline	2.94 (3.81)*	2.26 (2.86)	2.42 (2.21)*	***0.047***	0.90
	3rd month	2.88 (3.49)	1.99 (1.53)	1.87 (2.36)	0.17	
	P time	0.15	0.64	0.31		
cSBPoffice (mmHg)	Baseline	112.38 ± 10.26	112.37 ± 10.38	111.96 ± 9.08	0.99	0.66
	3rd month	109.75 ± 9.90	112.55 ± 11.80	115.68 ± 13.65	0.27	
	P time	0.31	0.84	0.29		
cDBPoffice (mmHg)	Baseline	76.5 ± 7.10	77.89 ± 7.97	75.13 ± 8.99	0.54	0.92
	3rd month	71 (8)*	79 (13)	74 (9)*	***0.016***	
	P time	***0.002***	0.98	0.74		
PWVoffice (m/s)	Baseline	9 (2.75)	9.25 (1.93)	10.3 (1.75)	0.14	0.90
	3rd month	9.53 ± 1.35	9.37 ± 1.21	10.43 ± 1.55	***0.05***	
	P time	***0.049***	0.81	0.79		

Statistical methods as in Supplementary Table 2. FMD, flow-mediated dilatation; SBP, systolic blood pressure; DBP, diastolic blood pressure; cSBP/cDBP, central systolic/diastolic blood pressure; PWV, pulse wave velocity.

Supplementary Table 13. BMI and blood biochemical measurements in non-CAD participants.

Variable	Time	Placebo (N=17)	Low dose (N=11)	High dose (N=11)	P groups	P time*group
BMI (kg/m²)	Baseline	28.52 ± 4.36	30.13 ± 3.83	29.41 ± 4.98	0.47	0.19
	3rd month	27.97 (4.7)	28.89 (7)	27.27 (6.5)	0.67	
	P time	0.68	0.66	0.27		
Glu (mg/dL)	Baseline	94 (14)	93 (12)	95 (15)	0.92	0.17
	3rd month	93 (12)	95 (20)	96 (14)	0.07	
	P time	***0.042***	0.79	0.09		
TC (mg/dL)	Baseline	158.5 (52)	169 (56)	164 (81)	0.89	0.67
	3rd month	166.25 ± 34.62	174.42 ± 35.96	175.46 ± 43.72	0.64	
	P time	0.39	0.17	0.09		
TG (mg/dL)	Baseline	103.5 (52)	95 (61)	76.5 (50)	0.99	***0.015***
	3rd month	108.5 (42)	108 (40)	81 (56)	0.70	
	P time	0.79	0.14	0.32		
HDL (mg/dL)	Baseline	57.96 ± 12.73	48.05 ± 12.77	55.08 ± 15.17	0.48	0.19
	3rd month	57.5 ± 11.75	48.32 ± 13.98	54.46 ± 14.44	0.07	

Variable	Time	Placebo (N=17)	Low dose (N=11)	High dose (N=11)	P groups	P time*group
	P time	0.79	0.41	0.71		
LDL (mg/dL)	Baseline	87.5 (49)	108 (65)	87 (49)	0.53	0.68
	3rd month	92.04 ± 31.95	107.89 ± 33.93	104.92 ± 41.31	0.26	
	P time	0.44	0.32	0.08		
CRP (mg/L)	Baseline	1.16 (1.46)	2.45 (3.9)	1.37 (2.27)	0.64	***<0.001***
	3rd month	0.89 (2.5)	1.63 (5.56)	1.17 (1.44)	0.30	
	P time	0.23	0.37	0.96		

Statistical methods as in Supplementary Table 2. BMI, body mass index; Glu, glucose; TC, total cholesterol; TG, triglycerides; HDL, high-density lipoprotein cholesterol; LDL, low-density lipoprotein cholesterol; CRP, C-reactive protein.

Supplementary References

1. Pachi VK, Mikropoulou E V., Gkiouvetidis P, Siafakas K, Argyropoulou A, Angelis A, et al. Traditional uses, phytochemistry and pharmacology of Chios mastic gum (Pistacia lentiscus var. Chia, Anacardiaceae): A review. Journal of Ethnopharmacology. Elsevier Ireland Ltd; 2020. doi:10.1016/j.jep.2019.112485 PubMed PMID: 32092498.
2. Svingou D, Mikropoulou E V., Pachi VK, Smyrnioudis I, Halabalaki M. Chios mastic gum: A validated method towards authentication. Journal of Food Composition and Analysis. 2023 Jan 1;115. doi:10.1016/j.jfca.2022.104997
3. Kim D, Langmead B, Salzberg SL. HISAT: A fast spliced aligner with low memory requirements. Nat Methods. 2015 Mar 31;12(4):357–60. doi:10.1038/nmeth.3317 PubMed PMID: 25751142.
4. Anders S, Pyl PT, Huber W. HTSeq-A Python framework to work with high-throughput sequencing data. Bioinformatics. 2015 Jan 15;31(2):166–9. doi:10.1093/bioinformatics/btu638 PubMed PMID: 25260700.
5. Love MI, Huber W, Anders S. Moderated estimation of fold change and dispersion for RNA-seq data with DESeq2. Genome Biol. 2014 Dec 5;15(12). doi:10.1186/s13059-014-0550-8 PubMed PMID: 25516281.
6. Kuleshov M V., Jones MR, Rouillard AD, Fernandez NF, Duan Q, Wang Z, et al. Enrichr: a comprehensive gene set enrichment analysis web server 2016 update. Nucleic Acids Res. 2016 Jul 8;44(1):W90–7. doi:10.1093/nar/gkw377 PubMed PMID: 27141961.
